# Ostéochondrome volumineux de l'omoplate: à propos d'un cas

**DOI:** 10.11604/pamj.2015.22.360.8305

**Published:** 2015-12-11

**Authors:** Mohamed Amine Karabila, Leila Otmani, Mohamed Azouz, Younes Mhamdi, Ismail Hmouri, Mohamed Kharmaz, Ahmed Bardouni, Abdou Lahlou, Mustapha Mahfoud, Mohamed Salehberrada

**Affiliations:** 1 Service de Chirurgie Orthopédique et de Traumatologie, CHU Ibn Sina, Rabat, Maroc

**Keywords:** Omoplate, ostéochondomatose, volumineux, Scapula, osteochondomatosis, large

## Abstract

Nous rapportons le cas d'un ostéochondrome de l'omoplate ayant un aspect radiologique et une localisation inhabituelle chez un jeune homme de 24 ans qui consulte pour une déformation de son épaule gauche avec une bosse postérieure refoulant son omoplate. Un bilan radiologique standard a montré une exostose pédiculée à la partie supérieure de l'omoplate. La tomodensitométrie était en faveur d'une exostose volumineuse. La résection chirurgicale de la tumeur et l'examen anatomo-pathologique ont permis de confirmer le diagnostic d'un ostéochondrome. Après un recul de dix mois, les résultats fonctionnels étaient très bons.

## Introduction

L'exostose appelée aussi ostéochondrome, correspond à une excroissance osseuse recouverte d'une coiffe cartilagineuse [1]. Elle est la plus fréquente des tumeurs bénignes de l'os et représente environ 35% de l'ensemble des tumeurs osseuses bénignes. Dans la majorité des cas, elle est localisée aux épiphyses des os longs, surtout le fémur et l'humérus. La localisation à l'omoplate est rare, en particulier sur la face dorsale.

## Patient et observation

**Figure 1:**
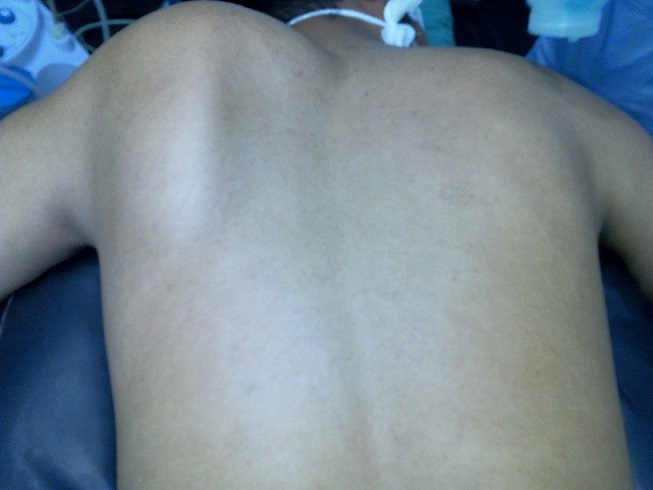
Aspect clinique de la tumeur refoulant l'omoplate en arrière

**Figure 2:**
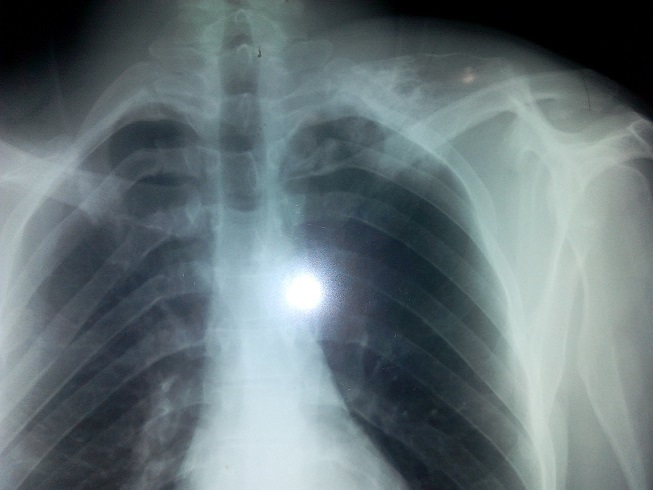
Aspect radiologique de l'ostéochondrome pédiculé au bord spinal de l'omoplate

**Figure 3:**
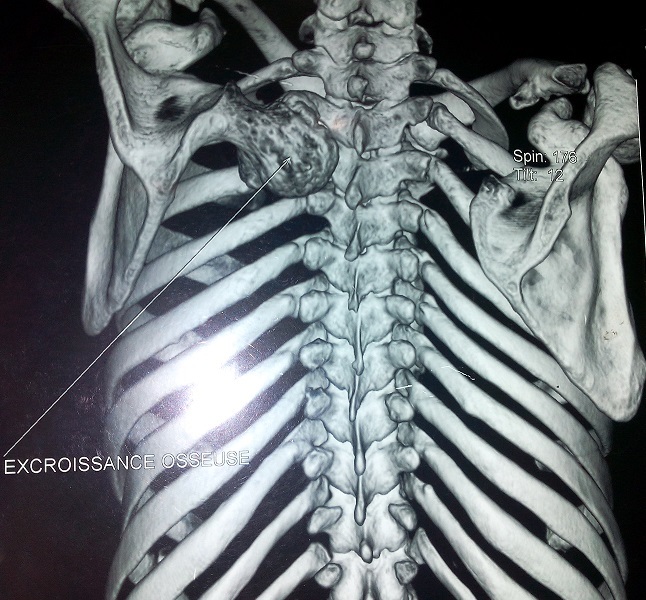
Aspect scannographique de l'ostéchondrome de l'omoplate

**Figure 4:**
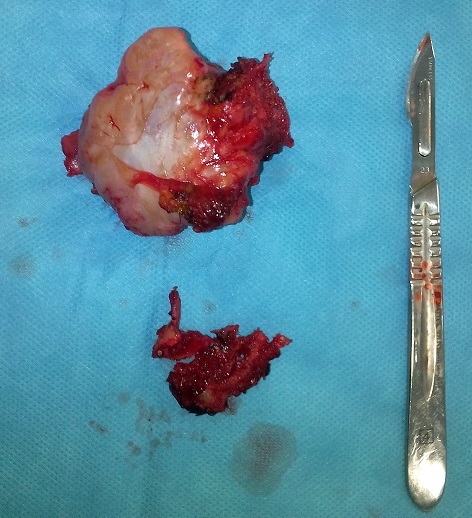
Aspect macroscopique de l'ostéochondrome

Un jeune homme de 24 ans été présenté à la consultation pour une masse de la face dorsale de l'omoplate évoluant depuis 3 ans. Cette masse était dure, non douloureuse et légèrement mobile; l'omoplate formait une volumineuse bosse dorsale de 12 cm de long, 8 cm de large et 5 cm d’épaisseur (Figure 1). Cette masse entraînait une fatigabilité de l’épaule à l'effort, une limitation des mouvements articulaires de l’épaule surtout actifs et un grand désagrément esthétique. La radiographie standard de l'omoplate de face a objectivé une formation osseuse hétérogène du bord spinal de l'omoplate (Figure 2). Un scanner a confirmé la présence de la volumineuse exostose, dont les limites apparaissent régulières et corticalisées (Figure 3). Le patient a été opéré, avec une résection en bloc de la tumeur et extraction des fragments détachés (Figure 4). L'examen anatomopathologique a conclu un ostéochondrome. En post opératoire, le patient a bénéficié d'un programme de rééducation visant l'obtention d'une indolence, la récupération des amplitudes articulaires et de la force des muscles de l’épaule. A six mois post-opératoire, le patient a repris son travail sans incident et aucune récidive n'a été constatée après 10 mois de recul.

## Discussion

L'ostéochondrome constitue 35% des tumeurs osseuses bénignes et 8,5% de l'ensemble des tumeurs osseuses [2]. Elles surviennent souvent chez les adolescents et rarement chez les nouveau-nés. On connait deux formes cliniques: les exostoses solitaires et la maladie exostosante [3, 4]. Pour l'exostose solitaire, il n'y a pas de différence entre les deux sexes. Par contre, la maladie exostosante affecte plus fréquemment les hommes. Elle touche le plus souvent les métaphyses des os longs (fémur, humérus et tibia). Ces zones ont l'activité de croissance osseuse la plus importante. La localisation aux os plats est possible, mais reste très rare. Au niveau de l'omoplate, l'ostéochondrome est la tumeur primitive la plus commune, avec une incidence de 4,6%. Elle est le plus souvent localisée à la face antérieure de l'os, ce qui entraîne des complications de type mécanique [5]. Ainsi H. Tomo et al. ont rapporté un cas de déformation de la cage thoracique consécutif à un ostéochondrome de la face antérieure de l'omoplate [6]. D'autres complications ont été rapportées dans la littérature: la plus fréquemment citée est la bursite, qui se manifeste par une douleur et une diminution de la mobilité de l’épaule [7]. Le diagnostic d'ostéochondrome est facile à poser, car les données de l'imagerie radiologique standard sont suffisantes pour confirmer le diagnostic. Néanmoins, dans des rares cas, du fait de la localisation, du volume ou de l'aspect radiologique atypique de la tumeur, le recours à la tomodensitométrie, voire à une biopsie, se révèle nécessaire. Chez notre patiente, le volume et l'aspect radiologique de la tumeur ainsi que le jeune âge ont justifié une biopsie chirurgicale avant exérèse totale de la tumeur. Le traitement chirurgical est indiqué en cas d'ostéochondrome symptomatique, gênant esthétiquement, ou en cas de suspicion de malignité. L'exérèse doit être faite le plus complètement possible tout en préservant au maximum l'intégrité de la pièce osseuse, siège de la lésion. Le risque de dégénérescence est de 1-2% dans l'exostose solitaire [8] et de 10 à 20% dans la maladie exostosante [9]. Le risque de transformation maligne est très faible en cas de lésion unique (1 à 2% des cas). Ce risque est de 5 à 25% en cas de lésions multiples. Dans la majorité des cas, le pronostic après chirurgie est excellent, avec une disparition rapide de la symptomatologie. L'apparition de certains signes doivent faire redouter une transformation maligne, à savoir l'augmentation de la taille de la tumeur; l'apparition d'une ostéolyse; l'aspect flou des bords de l'exostose; la présence de calcifications en dehors de l'ossification principale; l’érosion de l'os porteur ou de l'os voisin; une épaisseur de plus de 1cm de la coiffe de cartilage et l'hyperfixation scintigraphique chez l'adulte. La présence d'un seul de ces signes doit conduire à une exérèse de type carcinologique.

## Conclusion

L'ostéochondrome est une tumeur bénigne fréquente et connue, mais, dans certains cas rares, elle peut être trompeuse du fait de sa localisation ou de son aspect radiologique atypiques. Le risque de dégénérescence sarcomateuse nécessite une surveillance clinique et radiologique rapprochée et une exérèse chirurgicale au moindre doute.

## References

[1] Tomeno B (2000). Tumeurs cartilagineuses bénignes.

[2] Dahlin DC, Unni KK (1986). Bone tumors: General aspects and data on 8,542 cases.

[3] Gouin F, Venet G, Moreau A (2001). Exostoses solitaires, maladies exostosantes et autres exostoses-Encyclopé-die Médico Chirurgicale. Traité de l'appareil locomoteur..

[4] Lee KCY, Davies AM, Cassar-Puillicino VN (2002). Imaging the complications of osteochondroma. Clin Radiol..

[5] Percy EC, Birbrager D, Pitt MJ (1988). Snapping scapula: a review of the literature and presentation of 14 patients. Can J Surg..

[6] Tomo H, Ito Y, Aono M, Takaoka KD (2005). Chest wall deformity associated with osteochondroma of the scapula: a case report and review of the literature. J Shoulder Elbow Surg..

[7] Gamanagatti S, Gugalani B, Singh N (2004). Large bursa associated with osteochondroma of ventral surface of scapula. Europ J Radiol..

[8] Willms R, Hartwig CH, Bohm P, Sell S (1997). Malignant transformation of a multiple cartilaginousexostosis: a case report. Int Orthop..

[9] Poey C, Clement JL (1991). Ostéochondrome. EMC: Radiodiagnostic-Neurologie-Appareil locomoteur.

